# Nonalcoholic fatty liver disease, circulating ketone bodies and all‐cause mortality in a general population‐based cohort

**DOI:** 10.1111/eci.13627

**Published:** 2021-06-13

**Authors:** Adrian Post, Erwin Garcia, Eline H. van den Berg, Jose L. Flores‐Guerrero, Eke G. Gruppen, Dion Groothof, Berend Daan Westenbrink, Margery A. Connelly, Stephan J.L. Bakker, Robin P. F. Dullaart

**Affiliations:** ^1^ Department of Internal Medicine University Medical Center Groningen University of Groningen Groningen The Netherlands; ^2^ Laboratory Corporation of America Holdings (Labcorp) Morrisville NC USA; ^3^ Department of Gastroenterology and Hepatology University Medical Center Groningen University of Groningen Groningen The Netherlands; ^4^ Department of Cardiology University Medical Center Groningen University of Groningen Groningen The Netherlands

**Keywords:** general population, ketone bodies, mortality, nonalcoholic fatty liver disease

## Abstract

**Background:**

Nonalcoholic fatty liver disease (NAFLD) is increasingly prevalent, paralleling the obesity epidemic. Ketone bodies are produced in the liver, but it is currently uncertain whether circulating ketone bodies are increased in the context of NAFLD. We investigated the association between NAFLD and circulating ketone bodies and determined the extent to which NAFLD and circulating ketone bodies are associated with all‐cause mortality.

**Methods:**

Plasma ketone bodies were measured by nuclear magnetic resonance spectroscopy in participants of the general population‐based PREVEND study. A fatty liver index (FLI) ≥60 was regarded as a proxy of NAFLD. Associations of an elevated FLI and ketone bodies with all‐cause mortality were investigated using Cox regression analyses.

**Results:**

The study included 6,297 participants aged 54 ± 12 years, of whom 1,970 (31%) had elevated FLI. Participants with elevated FLI had higher total ketone bodies (194 [153‐259] vs 170 [133‐243] µmol/L; *P* < .001) than participants without elevated FLI. During 7.9 [7.8‐8.9] years of follow‐up, 387 (6%) participants died. An elevated FLI was independently associated with an increased risk of mortality (HR: 1.34 [1.06‐1.70]; *P* = .02). Higher total ketone bodies were also associated with an increased mortality risk (HR per doubling: 1.29 [1.12‐1.49]; *P* < .001). Mediation analysis suggested that the association of elevated FLI with all‐cause mortality was in part mediated by ketone bodies (proportion mediated: 10%, *P* < .001).

**Conclusion:**

Circulating ketone bodies were increased in participants with suspected NAFLD. Both suspected NAFLD and higher circulating ketone bodies are associated with an increased risk of all‐cause mortality.

## 1 INTRODUCTION

Nonalcoholic fatty liver disease (NAFLD), defined as hepatic steatosis in the absence of excessive alcohol use, is becoming the most common chronic liver disease in the Western world.^1^ The prevalence of NAFLD has markedly increased over the last years, concomitantly with increases in the prevalence of both obesity and the metabolic syndrome.^2^ The global prevalence of NAFLD is currently estimated to be 25% with the highest prevalence in the Middle East and South America and the lowest in Africa.^3^ Using an elevated fatty liver index (FLI), a validated proxy of NAFLD,^4^, ^5^ a similar prevalence of NAFLD was reported in a large North European cohort.^6^ As NAFLD progresses, hepatic inflammation and hepatic fibrosis develop, increasing the risk of cirrhosis, liver failure and hepatocellular carcinoma. Furthermore, NAFLD is linked to multiple cardio‐metabolic disorders including type 2 diabetes (T2D), cardiovascular disease, hypertension, chronic kidney disease and cardiac arrhythmias.^7^, ^8^, ^9^ In line with the above, the presence of NAFLD has been associated with an increased risk of mortality.^10^, ^11^, ^12^, ^13^

Human metabolism is characterized by great flexibility, allowing it to utilize different metabolic substrates depending of their availability to obtain energy.^14^ Ketogenesis is a liver‐bound metabolic process taking place in the mitochondria of perivenous hepatocytes in which fatty acids are converted into ketone bodies, consisting of β‐hydroxybutyrate, acetoacetate and acetone.^14^, ^15^ Fatty acid β‐oxidation leads to the production of acetyl‐CoA, which can be either used in the citric acid cycle or it can be subsequently converted to acetoacetyl‐CoA, 3‐hydroxy‐3‐methylglutaryl‐CoA (HMG‐CoA) and eventually into acetoacetate, from which β‐hydroxybutyrate and acetone are derived.^15^ The combination of a high intrahepatic fatty acid content and insulin resistance may predispose patients with NAFLD to increased ketogenesis by providing more substrate for ketone body production.

Data regarding the effects of hepatic steatosis on circulating ketone bodies are inconclusive, with increases,^16^, ^17^ decreases ^18^, ^19^, ^20^ and similar plasma concentrations being described in literature.^21^ To our knowledge, no large population‐based cohort studies have assessed the association of fasting circulating ketone body concentrations with NAFLD.

The aim of the present study was to determine whether circulating ketone bodies are increased in the context of FLI‐assessed NAFLD in a large population‐based cohort. Furthermore, the associations of FLI‐assessed NAFLD and ketone bodies with all‐cause mortality were longitudinally investigated.

## 2 METHODS

### 2.1 Study design and participants

This study was conducted as part of the Prevention of REnal and Vascular ENd‐stage Disease (PREVEND) study, a prospective Dutch cohort study in a general population, among inhabitants, aged between 28 and 75 years of the city Groningen, the Netherlands. The details of the study design and recruitment have been described before.^22^ We excluded participants with no data on the fatty liver index (FLI) and participants with no data on plasma ketone bodies, leaving a total of 6,297 participants for the current study. Detailed information on the flow of participants through the study is provided in Figure S1. The PREVEND study has been approved by the local medical ethics committee (approval number: MEC96/01/022) and was undertaken in accordance with the Declaration of Helsinki. All participants provided written informed consent. Reporting of the study conforms to broad EQUATOR guidelines.^23^

The second screening round comprised two visits to an outpatient clinic separated by three weeks. Self‐administered questionnaires concerning demographics, cardiovascular and renal disease history, smoking habits and medication use were provided by all participants prior to the first visit. Information on medication use was combined with information from IADB.nl, a database containing information of prescribed medication in public pharmacies in the Netherlands since 1999 (http://www.iadb.nl/).

Height and weight were measured with the participants standing without shoes and heavy outer garments. Body mass index (BMI) was calculated by dividing weight in kilograms by height, in metres, squared. Waist circumference was measured as the smallest girth between the rib cage and iliac crest.

### 2.2 Mortality Outcome

Follow‐up time was defined as the period between the second screening round (baseline) and events defined as death, loss to follow‐up or the end of follow‐up time (01‐01‐2011), whichever came first. If a person had moved to an unknown destination, the date on which the person was dropped from the municipal registry was used as the census date. The primary outcome for the study was all‐cause mortality. Secondarily, we performed analyses on cardiovascular mortality, noncardiovascular mortality and cancer mortality. Data on mortality were obtained from the municipal register, and the cause of death was obtained by linking the number of the death certificate to the primary cause of death as coded by a physician from the Central Bureau of Statistics, according to the 10th revision of the International Classification of Diseases.

### 2.3 NAFLD assessment

For the diagnosis of NAFLD, the algorithm of the fatty liver index (FLI) was used. The FLI was calculated as follows:

$$\text{FLI}=\frac{e^{\left(0.953*\text{loge}(\text{triglycerides})+0.139*\text{BMI}+0.718*\text{loge}(\text{ggt})+0.053*\text{waist circumfernce}‐15.745\right)}}{1+e^{\left(0.953*\text{loge}(\text{triglycerides})+0.139*\text{BMI}+0.718*\text{loge}(\text{ggt})+0.053*\text{waist circumfernce}‐15.745\right)}}*100.$$

where GGT is gamma‐glutamyltransferase. The optimal cut‐off value for the FLI has been documented to be 60 with an accuracy of 84%, a sensitivity of 61% and a specificity of 86% for detecting suspected NAFLD as determined by ultrasonography.^5^ The FLI is currently considered as one of the best‐validated steatosis scores for larger scale screening studies.^4^ Participants with a FLI score ≥60 were classified as having NAFLD.

### 2.4 Laboratory measurements

Baseline venous blood samples were drawn between 8:00 and 10:00 am from all participants after an instructed overnight fast. Aliquots of ethylenediaminetetraacetic acid (EDTA) plasma were obtained by centrifugation, and samples were immediately frozen at −80℃ until analysis.

Total ketone bodies were calculated as the sum of β‐hydroxybutyrate, acetoacetate and acetone, which were measured in EDTA‐plasma samples using a Vantera Clinical Analyzer (Labcorp, Morrisville, NC), a fully automated, high‐throughput, 400 MHz proton (1H) nuclear magnetic resonance (NMR) spectroscopy platform. A detailed description of the data acquisition and method validation is described elsewhere.^24^ In brief, a method comparison study was performed comparing quantification by NMR to platforms commonly used for determining ketone body concentrations, that is LC/MS/MS for β‐hydroxybutyrate and acetoacetate and GC/MS for acetone. A comparison of plasma concentrations using the comparator platforms correlated well by Deming regression with R^2^ values of 0.996, 0.994 and 0.994 for β‐hydroxybutyrate, acetoacetate and acetone, respectively. For β‐hydroxybutyrate, acetoacetate and acetone, coefficients of variation for intra‐assay and inter‐assay precision were 1.3%–9.3%, 3.1%–7.7%, and 3.8%–9.1%, respectively.

Serum alanine aminotransferase (ALT) and aspartate aminotransferase (AST) were measured using the standardized kinetic method with pyridoxal phosphate activation (Roche Modular P, Roche Diagnostics, Mannheim, Germany). Serum GGT was assayed by an enzymatic colorimetric method (Roche Modular P, Roche Diagnostics, Mannheim, Germany). Standardization of ALT, AST and GGT was performed according to the International Federation of Clinical Chemistry guidelines. Serum creatinine was measured with an enzymatic method on a Roche Modular analyzer, using reagents and calibrators from Roche (Roche Diagnostics, Mannheim, Germany). Fasting plasma glucose was measured by dry chemistry (Eastman Kodak, Rochester, NY, USA). Total cholesterol, HDL cholesterol triglycerides, insulin and serum cystatin C were measured using standard protocols, which have been described previously.^25^, ^26^, ^27^, ^28^ Estimated glomerular filtration rate (eGFR) was calculated using the Chronic Kidney Disease Epidemiology Collaboration (CKD‐EPI) combined creatinine‐cystatin C equation.^29^ N‐terminal pro‐B‐type natriuretic peptide (NT‐proBNP) was assayed in plasma using a Elecsys™ 2010 analyzer.

### 2.5 Statistical analyses

Statistical analyses were performed with R version 3.6.2 (Vienna, Austria) (http://cran.r‐project.org/). Results were expressed as mean ± standard deviation (SD), median [interquartile range] or number (percentage) for normally distributed, skewed and categorical data, respectively. A two‐sided *P‐*value <.05 was considered to indicate statistical significance. Comparisons of baseline characteristics and circulating ketone bodies between participants with and without NAFLD were tested using independent sample t test, Mann‐Whitney U test or chi‐square test where appropriate. Additionally, the differences in ketone bodies were graphically represented using a bar plot with error bars representing the 95% confidence interval.

Associations between circulating ketone bodies and elevated FLI were also investigated using multivariable logistic regression analyses, in which adjustments were made for the potential confounders age, sex, alcohol intake, smoking status, total cholesterol, HDL cholesterol, systolic blood pressure, NT‐ProBNP, eGFR, albuminuria, type 2 diabetes (T2D), glucose, insulin and use of glucose‐lowering drugs.

Cox proportional hazards models were used to investigate the associations of elevated FLI and total ketone bodies with all‐cause mortality. Hazard ratios were computed per doubling of total ketone bodies. Deviations from linearity were tested by comparing linear models with nonlinear models using natural cubic splines with two degrees of freedom. The proportional hazards assumption was verified visually with plots of the scaled Schoenfeld residuals and was not violated in any of the models. Adjustments were made for a priori selected variables, including age, sex, smoking, alcohol intake, total cholesterol, HDL cholesterol, systolic blood pressure, NT‐ProBNP, eGFR, urinary albumin excretion, history of cardiovascular disease, history of malignancy, T2D, glucose, insulin and use of glucose‐lowering drugs. Interactions of elevated FLI or total ketone bodies with the potential confounders were tested by including interaction terms in the model. A *P*‐interaction <.10 was considered to indicate significant effect modification.^30^ Stratified Cox proportional hazards regression analyses were performed to assess the association of NAFLD and total ketone bodies with the risk of all‐cause mortality according to significant effect modifiers. For nonordinal variables, stratification was performed over the median in the deceased group, thereby allowing a similar number of events among the groups.

Mediation analyses were used to investigate whether the association of elevated FLI with all‐cause mortality was mediated through circulating ketone bodies. Mediation analyses was performed using the mediation package in R, according to the method as described by Preacher and Hayes,^31^ which allowed for testing the significance and magnitude of (potential) mediation. In these analyses, mediation was assessed upon running 2000 Monte Carlo draws for quasi‐Bayesian approximation. The proportion of mediation was obtained by dividing the standardized indirect effect coefficient by the standardized total effect coefficient, which were in each model adjusted for age and sex.

To visualize the continuous associations of elevated FLI with all‐cause mortality, a Kaplan‐Meier curve was generated. To visualize the association of circulating total ketone bodies with all‐cause mortality, circulating total ketone bodies, as a continuous variable, was plotted against the risk of all‐cause mortality.

Sensitivity analyses were conducted to evaluate the robustness of the findings, wherein any potential bias caused by the presence of T2D was accounted for by excluding participants with T2D at baseline. Given the strong predictive value of previous cardiovascular events, an additional sensitivity analysis was performed in which participants with a history of cardiovascular disease were excluded. Furthermore, to account for possible effects of obesity or underweight, sensitivity analyses were performed in which participants with a BMI >30 kg/m^2^ or <18.5 kg/m^2^ were excluded.^32^ Also, a sensitivity analysis was performed in which participants with an alcohol intake ≥1 consumption per day were excluded. Lastly, to account for potential bias introduced by reverse causation, a sensitivity analysis was performed in which we excluded the first two years of follow‐up.

## 3 RESULTS

### 3.1 Baseline characteristics

**FIGURE 1:**
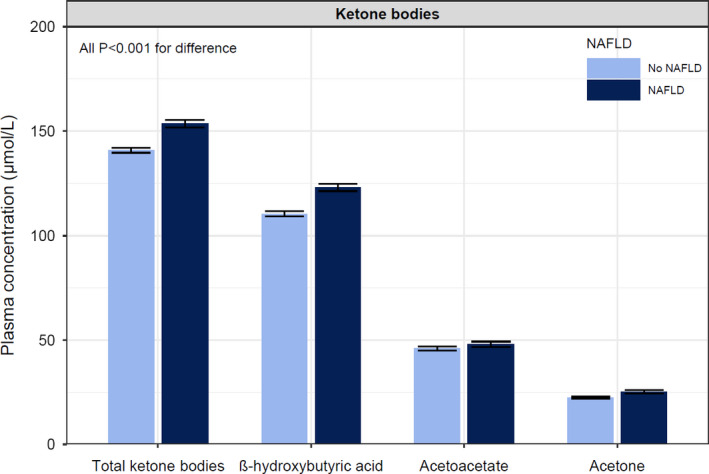
Barplot displaying the differences in ketone bodies among participants with and without suspected NAFLD based on an elevated FLI. The error bars represent the 95% confidence interval. Participants with NAFLD had higher total ketone bodies (194 [153‐259] vs 170 [133‐243] µmol/L; *P* < .001), β‐hydroxybutyric acid (133 [104‐179] vs 116 [89‐165] µmol/L; *P* < .001), acetoacetate (41 [28‐59] vs 38 [25‐57] µmol/L; *P* < .001) and acetone (22 [14‐32] vs 19 [12‐28] µmol/L; *P* < .001) than participants without NAFLD

Among the 6,297 participants aged 54 ± 12 years, 1,970 (31%) were classified with NAFLD based on an FLI ≥ 60. An overview of baseline characteristics according to NAFLD status, that is a FLI ≥ 60, is shown in Table 1. Participants with an elevated FLI had higher total ketone bodies (194 [153‐259] vs 170 [133‐243] µmol/L; *P* < .001) than participants without an elevated FLI. Individual ketone bodies, β‐hydroxybutyrate, acetoacetate and acetone were all higher too (*P* < .001 for each). A barplot of circulating ketone concentrations is shown in Figure 1. In addition, the ratio of acetone to acetoacetate was higher in participants with NAFLD (0.51 [0.34‐0.76]) as compared to the ratio in participants without NAFLD (0.48 [0.32‐0.70]), *P* < .001) (Table S1).

**TABLE 1:** Baseline clinical and laboratory characteristics according to suspected NAFLD (fatty liver index (FLI) ≥60)

Variables	No NAFLD (FLI <60)	NAFLD (FLI ≥60)	*P*‐value
Participants, n	4,327	1,970	<.001
Sex, n (%) male	1,820 (42)	1,308 (66)	<.001
Age, years	52 ± 12	57 ± 11	<.001
Type 2 diabetes, n (%)	127 (3)	256 (13)	<.001
Hypertension, n (%)	1,021 (24)	1,077 (55)	<.001
History of cardiovascular disease, n (%)	192 (4)	199 (10)	<.001
History of malignancy, n (%)	249 (6)	104 (5)	.50
BMI, kg/m^2^	24.8 ± 2.8	30.9 ± 4.1	<.001
Waist circumference, cm	86 ± 9	105 ± 9	<.001
Systolic blood pressure, mmHg	122 ± 17	135 ± 18	<.001
Diastolic blood pressure, mmHg	72 ± 9	77 ± 9	<.001
NT‐ProBNP, ng/L	43 [23‐82]	37 [17‐78]	<.001
Antihypertensive treatment, n (%)	567 (15)	666 (37)	<.001
Smoking status, current n (%)	1235 (29)	519 (27)	.07
Alcohol intake			
No, almost never, n (%)	1,015 (24)	560 (29)	<.001
<1 drink per day, n (%)	2,171 (51)	850 (44)	
≥1 drink per day, n (%)	1,097 (26)	544 (28)	
Total cholesterol, mmol/L	5.3 ± 1.0	5.7 ± 1.1	<.001
HDL cholesterol, mmol/L	1.3 ± 0.3	1.1 ± 0.2	<.001
Triglycerides, mmol/L	0.95 [0.72‐1.27]	1.71 [1.30‐2.34]	<.001
Lipid‐lowering drugs, n (%)	232 (6)	266 (15)	<.001
ALT, U/L	15 [12‐20]	23 [17‐32]	<.001
AST, U/L	21 [19‐25]	25 [21‐30]	<.001
Gamma‐GT, U/L	19 [14‐28]	41 [29‐63]	<.001
Glucose, mmol/L	4.7 [4.4‐5.1]	5.2 [4.7‐5.9]	<.001
Insulin, mU/L	6.9 [5.1‐9.3]	13.1 [9.5‐19.1]	<.001
Glucose‐lowering drugs, n (%)	72 (2)	152 (8)	<.001
eGFR, ml/min/1.73m^2^	94 ± 16	87 ± 18	<.001
Urinary albumin excretion, mg/24‐h	7.8 [5.8‐12.8]	11.7 [7.2‐27.0]	<.001
Ketone bodies			
Total ketone bodies, µmol/L	170 [133‐243]	194 [153‐259]	<.001
β‐hydroxybutyric acid, µmol/L	116 [89‐165]	133 [104‐179]	<.001
Acetoacetate, µmol/L	38 [25‐57]	41 [28‐59]	<.001
Acetone, µmol/L	19 [12‐28]	22 [14‐32]	<.001

Note

Comparisons of baseline characteristics and circulating ketone bodies between participants with and without NAFLD were tested using independent sample t test, Mann‐Whitney U test or chi‐square test where appropriate.

Participants with an elevated FLI were more likely to have higher age, T2D, a history of cardiovascular disease, a history of malignancy, higher blood pressure, lower NT‐ProBNP, lower alcohol intake, lower eGFR, higher urinary albumin excretion, higher glucose and higher insulin (all *P* < .05). Additionally, an overview of baseline characteristics according to sex‐stratified tertiles of total ketone bodies is shown in Table S2. Compared to the lowest sex‐stratified tertile, participants in the highest sex‐stratified tertile of total ketone bodies were more likely to be older, to have T2D, a history of cardiovascular disease, higher blood pressure, higher NT‐ProBNP and lower eGFR (all *P* < .05).

### 3.2 Logistic regression analyses

Univariable and multivariable logistic regression analyses of ketone bodies and an elevated FLI are shown in Table 2. Unadjusted, higher total ketone bodies were associated with higher odds of having an elevated FLI (OR per doubling: 1.31 [1.22‐1.41]; *P* < .001). After adjusting for age, sex, alcohol intake, smoking status, total cholesterol, HDL cholesterol, systolic blood pressure, NT‐ProBNP, eGFR and albuminuria, the association remained significant (OR: 1.23 [1.12‐1.36]; *P* < .001). Further adjustments for T2D or glucose, insulin and use of glucose‐lowering drugs did not materially change the association. No interaction with sex was found. Separate analyses of individual ketone bodies demonstrated that in univariable models, higher β‐hydroxybutyric acid, acetoacetate and acetone were each associated with higher odds of having an elevated FLI. However, multivariable analyses demonstrated that acetone was independently associated with an elevated FLI, whereas such associations with β‐hydroxybutyric acid and acetoacetate were lost in the multivariable models (Table 2). As a sensitivity analysis, we performed analyses in which we adjusted for waist circumference or BMI. Adjustment for waist circumference or BMI did not materially change the associations, with both total ketone bodies and acetone remaining significantly associated with an elevated FLI (Table S3).

**TABLE 2:** Multivariable logistic regression analyses of ketone bodies and suspected NAFLD (fatty liver index (FLI) ≥60)

	Total ketone bodies		β‐hydroxybutyric acid		Acetoacetate		Acetone	
	Odds ratio [95% CI]	*P*‐value	Odds ratio [95% CI]	*P*‐value	Odds ratio [95% CI]	*P*‐value	Odds ratio [95% CI]	*P*‐value
Model 1	1.31 [1.22‐1.41]	<.001	1.12 [1.05‐1.19]	<.001	1.12 [1.06‐1.18]	<.001	1.19 [1.13‐1.26]	<.001
Model 2	1.21 [1.12‐1.31]	<.001	1.07 [1.00‐1.13]	.04	1.03 [0.97‐1.09]	.3	1.12 [1.06‐1.18]	<.001
Model 3	1.23 [1.12‐1.36]	<.001	1.10 [1.02‐1.19]	.01	1.04 [0.97‐1.12]	.2	1.15 [1.08‐1.22]	<.001
Model 4a	1.20 [1.09‐1.32]	<.001	1.08 [1.01‐1.17]	.03	1.03 [0.96‐1.11]	.4	1.14 [1.07‐1.21]	<.001
Model 4b	1.20 [1.08‐1.34]	<.001	1.06 [0.97‐1.15]	.17	1.08 [1.00‐1.17]	.05	1.12 [1.05‐1.20]	.001

Note

Model 1: crude. Model 2: adjusted for age and sex. Model 3; as model 2, additionally adjusted for alcohol intake, smoking status, total cholesterol, HDL cholesterol, systolic blood pressure, NT‐ProBNP, eGFR and albuminuria (urinary albumin excretion >15 mg/24‐h). Model 4a: as model 3, additionally adjusted for type 2 diabetes. Model 4b: as model 3, additionally adjusted for glucose, insulin and use of glucose‐lowering drugs. All ketones are log_2_‐transformed for analyses.

### 3.3 Longitudinal analyses in the whole cohort

Among the 6,297 participants at risk, a total of 387 (6%) participants died during a follow‐up of 7.1 [3.6‐7.6] years. Participants who did not survive during follow‐up were more likely to have an elevated FLI (47% vs 30%; *P* < .001) and have higher circulating ketone bodies (213 [162‐314] µmol/L vs 176 [138‐246] µmol/L; *P* < .001) than participants who did survive. An overview of Cox regression analyses is shown in Table 3. Unadjusted, the presence of NAFLD was associated with an increased risk of all‐cause mortality (HR (95%): 2.00 [1.65‐2.45]; *P* < .001). In addition, higher circulating total ketone bodies were also associated with increased risk of all‐cause mortality (HR per doubling: 1.57 [1.39‐1.77]; *P* < .001).

**TABLE 3:** Prospective associations of suspected NAFLD (fatty liver index (FLI) ≥60) and ketone bodies with all‐cause mortality

	Suspected NAFLD vs. no NAFLD (FLI≥60 vs. FLI<60)		Total ketone bodies (Per doubling)	
	HR [95% CI]	*P*‐value	HR [95% CI]	*P*‐value
Model 1	2.00 [1.65‐2.45]	<.001	1.57 [1.39‐1.77]	<.001
Model 2	1.36 [1.11‐1.67]	.003	1.30 [1.14‐1.49]	<.001
Model 3	1.38 [1.09‐1.75]	.007	1.32 [1.15‐1.52]	<.001
Model 4a	1.34 [1.06‐1.70]	.02	1.29 [1.12‐1.49]	<.001
Model 4b	1.31 [1.01‐1.69]	.04	1.27 [1.10‐1.47]	.001
Model 5a	1.31 [1.04‐1.66]	.02	1.28 [1.11‐1.48]	<.001
Model 5b	1.27 [0.98‐1.64]	.07	1.26 [1.10‐1.47]	.001

Note

Models 1: crude. Models 2: adjusted for age and sex, current smoking and alcohol usage. Models 3: as models 2, additionally adjusted for total cholesterol, HDL cholesterol, systolic blood pressure, NT‐ProBNP, eGFR and urinary albumin excretion, history of cardiovascular disease and history of malignancy. Models 4a: as models 3, additionally adjusted for type 2 diabetes. Models 4b: as models 3, additionally adjusted for glucose, insulin and use of glucose‐lowering drugs. Model 5a: as models 4a, additionally adjusted for either suspected NAFLD or total ketone bodies. Models 5b: as models 4b, additionally adjusted for either suspected NAFLD or total ketone bodies.

After adjusting for age, sex, smoking, alcohol intake, total cholesterol, HDL cholesterol, systolic blood pressure, NT‐ProBNP, eGFR and urinary albumin excretion, both an elevated FLI (HR: 1.38 [1.09‐1.75]; *P* = .009) and higher circulating ketone bodies (HR: 1.32 [1.15‐1.52]; *P* < .001) remained significantly associated with an increased risk of all‐cause mortality. Further adjustment for potential confounders, including history of cardiovascular disease, history of malignancy, T2D, glucose, insulin and use of glucose‐lowering drugs, did not materially change the associations.

Concerning the association between NAFLD and all‐cause mortality, there was effect modification by smoking status (*P* = .07), eGFR (*P* = .05) and NT‐ProBNP (*P* = .08). Stratified analyses of the association between NAFLD and all‐cause mortality according to significant effect modifiers are shown in Tables S4‐S6. These analyses demonstrated that the association between NAFLD and all‐cause mortality was strongest in nonsmokers, participants with a lower eGFR and participants with a higher NT‐ProBNP. None of the other covariates was identified as a significant effect modifier (all *P* > .10).

In the association between total ketone bodies and all‐cause mortality, there was significant effect modification by history of malignancy (*P* < .001) and eGFR (*P* = .02). Stratified analyses of the association between total ketone bodies and all‐cause mortality according to significant effect modifiers are shown in Tables S7‐S8. These analyses demonstrated that the association between total ketone bodies and all‐cause mortality was strongest in participants with a history of malignancy and in participants with a lower eGFR. None of the other covariates was identified as a significant effect modifier (all *P* > .10).

**FIGURE 2:**
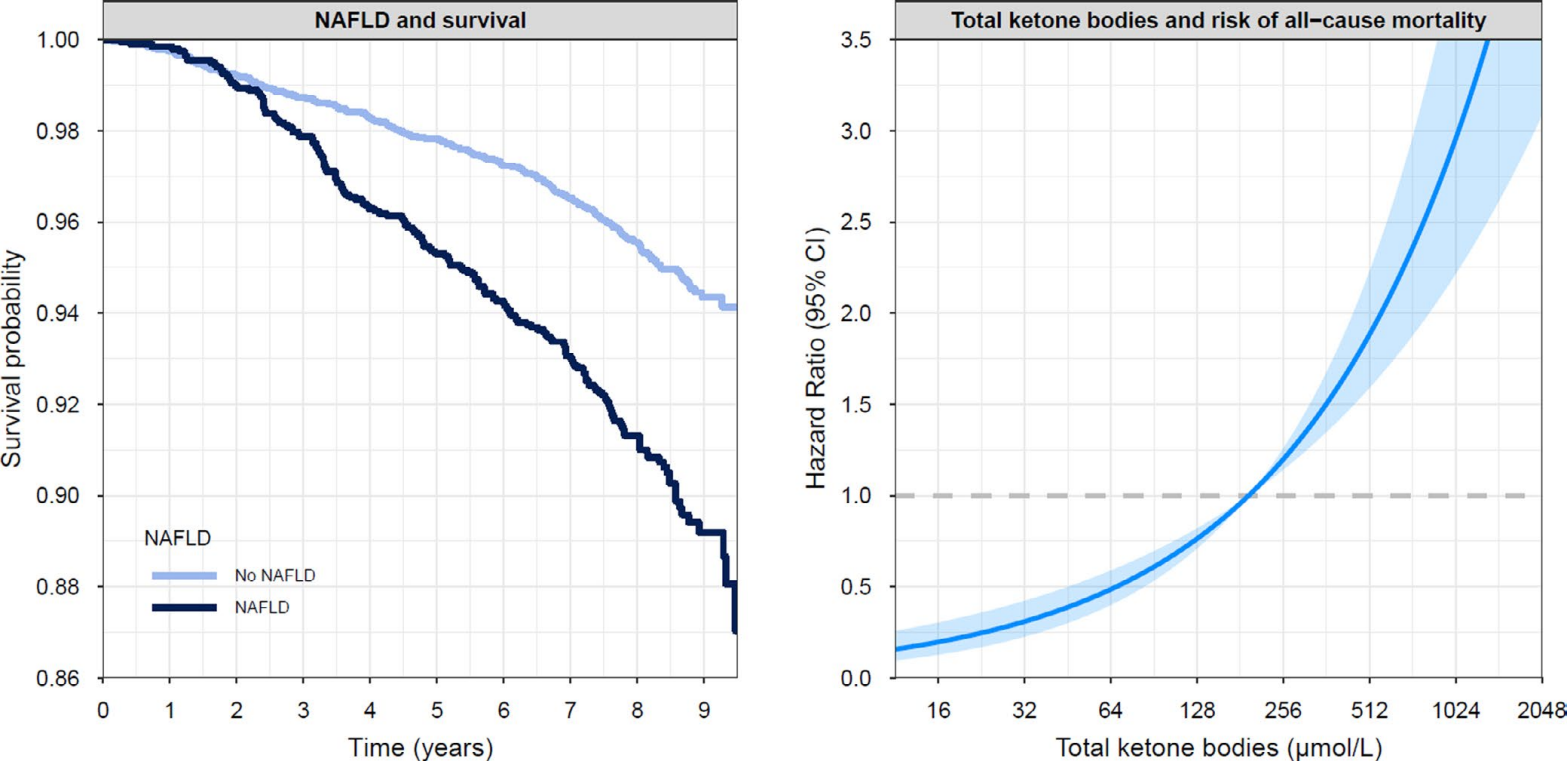
Kaplan‐Meier curve on the association between suspected NAFLD and all‐cause mortality (left) and a graphical representation of the association of circulating ketone bodies and the risk of all‐cause mortality (right). The line shows the hazard ratio (HR), and the shaded area corresponds to the 95% pointwise confidence interval (CI)

A graphical overview of the associations of an elevated FLI and circulating ketone bodies with all‐cause mortality is shown in Figure 2. Mediation analyses suggested that total ketone bodies explained 10% of the association found between an elevated FLI and all‐cause mortality (Table 4).

**TABLE 4:** Mediation analyses of the associations with all‐cause mortality

Predictor	Potential mediator	Effect (path)^a^	Multivariable model^b^		
			Coefficient [95% CI]	*P*‐value	Proportion mediated^c^
Suspected NAFLD	Ketone bodies	Indirect	0.001 [0.001‐0.002]	.001	10%
		Total	0.013 [0.001‐0.025]	.016	

^a^ For each path, the outcome is all‐cause mortality.

^b^ All coefficients are standardized coefficients with adjustments for age and sex.

^c^ The size of the significant mediated effect is calculated as the standardized indirect effect divided by the standardized total effect multiplied by 100.

Analyses of the individual ketones with all‐cause mortality demonstrated that the association of total ketone bodies with all‐cause mortality was primarily driven by β‐hydroxybutyric acid and acetoacetate. Higher β‐hydroxybutyric acid and higher acetoacetate were significantly associated with an increased risk of all‐cause mortality, independent of potential confounders (Table S9). Circulating acetone was not significantly associated with all‐cause mortality after adjustment for potential confounders.

In further analyses, it was assessed whether the prospective associations of elevated FLI and total ketone bodies were also present for cardiovascular mortality, noncardiovascular mortality and cancer mortality (Tables S10‐12). Among the 387 participants who died during follow‐up, 100 participants died due to cardiovascular mortality and 287 participants died due to noncardiovascular causes, of which 192 were due to cancer. In most adjusted models, elevated FLI was significantly associated with noncardiovascular mortality, but not significantly with cardiovascular mortality and cancer mortality. On the other hand, total ketone bodies were significantly associated with cardiovascular mortality, noncardiovascular mortality and cancer mortality in most adjusted models.

### 3.4 Sensitivity analyses

As sensitivity analyses, separate analyses were performed on the associations of elevated FLI and total ketone bodies with all‐cause mortality after excluding participants with T2D and participants with a history of cardiovascular disease. After excluding participants with T2D, the adjusted association of elevated FLI with all‐cause mortality lost significance, whereas the association of total ketone bodies with all‐cause mortality remained significant throughout the models (Table S13). After excluding participants with a history of cardiovascular disease, the adjusted association of elevated FLI with all‐cause mortality lost significance, whereas the association of total ketone bodies with all‐cause mortality remained significant throughout the models (Table S14).

We also performed sensitivity analyses after excluding participants with a BMI < 18.5 kg/m^2^. After excluding participants with a BMI < 18.5 kg/m^2^, the associations of both elevated FLI and total ketone bodies with all‐cause mortality remained significant except for an NAFLD model adjusted for glucose, insulin and use of glucose‐lowering drugs (Table S15). Similarly, we also performed sensitivity analyses after excluding participants with a BMI > 30 kg/m^2^. Excluding participants with a BMI > 30 kg/m^2^ did not materially change the associations (Table S16). Additionally, we performed sensitivity analyses in which excluded participants with an alcohol intake ≥1 consumption per day. After excluding participants with an alcohol intake ≥1 consumption per day, the association of elevated FLI with all‐cause mortality lost significance, whereas the association of total ketone bodies with all‐cause mortality remained significant throughout the models (Table S17).

Lastly, to account for potential bias introduced by reverse causation, a sensitivity analysis was performed in which we excluded the first two years of follow‐up. After excluding the first two years of follow‐up, the adjusted associations of elevated and total ketone bodies with all‐cause mortality remained significant (Table S18).

## 4 DISCUSSION

In this population‐based cohort, we have demonstrated to the best our knowledge for the first time that circulating ketone bodies are higher in subjects with suspected NAFLD, using an elevated FLI as proxy, as compared to those without an elevated FLI. In time‐to‐event analyses, both the presence of elevated FLI and circulating ketone bodies were independently associated with an increased risk of all‐cause mortality. Mediation analyses suggested that the association of suspected NAFLD with all‐cause mortality was in part mediated by higher circulating ketone bodies.

Ketone bodies are energy‐rich compounds that allow fat‐derived energy to be generated in the liver and used by other organs, such as the brain, heart, kidney and skeletal muscle when there is limited availability of carbohydrate or when carbohydrate cannot be used effectively. The process of ketogenesis takes place in the mitochondria of the perivenous hepatocytes and consists of β‐oxidation of fatty acids to acetyl‐CoA, the formation of acetoacetyl‐CoA, the conversion of acetoacetyl‐CoA to 3‐hydroxy‐3‐methylglutaryl‐CoA (HMG‐CoA) and, finally, conversion to acetoacetate, from which 3‐β‐hydroxybutyrate and acetone arise by reduction and spontaneous decarboxylation, respectively.^15^, ^33^ Ketogenesis occurs primarily in hepatic mitochondrial matrix at rates proportional to total fat oxidation.^34^ Key regulatory steps in ketogenesis include lipolysis of peripherally stored and circulating triglycerides, transport of nonesterified fatty acids to and across the hepatocyte plasma membrane, transport into mitochondria via allosterically regulated carnitine palmitoyltransferase 1, the rate of β‐oxidation and the hormonal regulators of these processes.^15^, ^33^ The major hormonal stimulator of ketogenesis is glucagon, and the major inhibitor of ketogenesis is insulin. Insulin inhibits the breakdown of triglycerides to fatty acids (hormone sensitive lipase) and inhibits HMG‐CoA synthase, providing one of the rate‐limiting steps in producing acetoacetate, the first ketone body in the series.^15^ Furthermore, insulin stimulates the conversion of acetyl‐CoA to malonyl‐CoA, a potent inhibitor of fatty acid transport into the mitochondria. In the current study, we demonstrated that circulating ketone body concentrations are higher in participants with an elevated FLI as compared to those without NAFLD. Insulin resistance is one of the pathophysiological hallmarks of NAFLD and, accordingly, plasma insulin levels were almost doubled in subjects with suspected NAFLD in the current study. Hence, higher circulating ketone body concentrations in NAFLD may at least in part be explained by impaired inhibition of ketogenesis by insulin. However, the association between circulating ketone bodies and the presence of an elevated FLI remained independent of adjustment for either T2D, glucose levels, insulin levels or usage of glucose‐lowering drugs, thereby implicating that the association may also in part be explained by other mechanisms. It has been established that in the setting of NAFLD, accumulation of hepatic triglycerides occurs due to increased de novo lipogenesis,^35^, ^36^ but also due to an increased delivery of nonesterified fatty acids from peripheral lipolysis,^37^ which may overall result in enhanced beta‐oxidation, leading to increased ketone production. A potential hypothesis is that ketogenesis is initially increased in NAFLD as a mechanism to safely manage excess triglyceride‐derived acetyl‐CoA via nonoxidative disposal to limit oxidative stress, a mechanism which becomes disturbed as NAFLD progresses. This notion is supported by a recent stable isotope tracer study in ketotic (24‐hour‐fasted) individuals with a wide range of hepatic triglyceride content levels (0%–52%), which demonstrated that ketogenesis was progressively impaired as hepatic steatosis worsened.^19^

The associations between the presence of NAFLD and mortality has equivocally been reported. While two earlier meta‐analyses did not find an association of NAFLD with all‐cause mortality,^3^, ^38^ a more recent and larger meta‐analysis that included 498 ,501 subjects (with 24, 234 deaths) did find an association between the presence of NAFLD and the risk of all‐cause mortality (HR: 1.34 [1.17‐1.54]).^13^ Furthermore, a recent study of 3, 003, 068 participants also demonstrated a significant association between NAFLD assessed by repeated measures of the FLI and the risk of all‐cause mortality.^39^ It is well established that cardiovascular disease prevalence is significantly associated with NAFLD and that NAFLD patients have a higher risk of developing cardiovascular disease than the general population.^8^ However, similar to the meta‐analysis of Lui *et al*, no significant association between NAFLD and cardiovascular mortality was found in the current study.^13^ This is line with a study in 9,200 participants who found a significant association between the fatty liver index and liver disease mortality, but no association with cardiovascular mortality.^40^ The potential mechanism behind this phenomenon remains uncertain. The authors of aforementioned meta‐analysis argued that due to their increase in cardiovascular risk factors, NAFLD patients could have been subject to more intense cardiovascular risk surveillance, thereby possibly counteracting their increased cardiovascular risk.

Of potential interest, a robust association between circulating ketone bodies and all‐cause mortality was observed. Ketones have primarily been studied in the context of ketogenic diets, which are able to lead to some improvements in cardiovascular risk factors, such as obesity, although these effects are usually limited in time.^41^ Furthermore, animal studies on ketogenic diets have also demonstrated a variety of neuroprotective, anti‐inflammatory and anti‐oxidant effects.^42^, ^43^, ^44^, ^45^ Nonetheless, in the current study, higher circulating ketone bodies were associated with an increased risk of all‐cause mortality. Although circulating ketone bodies have been associated with poor prognosis in specific patient populations,^46^, ^47^ to the best of our knowledge, it has not been investigated in the general population before. It is unknown whether circulating ketones are the culprit in this association or merely a reflection of an underlying mechanism.

It should be noted that the association found in the current study remained significant after adjusting for glucose, insulin, glucose‐lowering drugs and the presence of T2D, indicating that the association is not fully explained by alterations in insulin action. Furthermore, this association also remained significant after excluding participants with T2D as well as participants with a BMI < 18.5 or >30 kg/m^2^, implicating the association was not to an important extent explained by T2D, underweight or obesity. In an additional sensitivity analysis, the association remained significant after excluding the first two years of follow‐up, thereby accounting for potential bias that might have been introduced by reverse causation. Cause‐specific mortality analyses demonstrated that the association was strongest for noncardiovascular mortality and cancer mortality, and borderline significant for cardiovascular mortality. Yokokawa et al. demonstrated in a cohort of 615 patients hospitalized with heart failure that higher circulating acetoacetate levels were associated with an increased risk of all‐cause mortality.^46^ Furthermore, in patients with congestive heart failure, concentrations of circulating ketone bodies are increased in proportion to the severity of cardiac dysfunction.^48^ In heart failure, higher circulating ketone bodies are likely to be a reflection of the hypertrophied and failing heart shifting to ketone metabolism as a fuel for oxidative ATP production.^49^, ^50^ Interestingly, in a small crossover study, supplementation with 3‐hydroxybutyrate had beneficial hemodynamic effects in patients with heart failure and reduced ejection fraction.^51^

Obokata *et al* demonstrated in a cohort of 405 stable haemodialysis patients that higher circulating ketone bodies were associated with an increased risk of cardiovascular events and all‐cause mortality.^47^ It was speculated that an increased sympathetic tone may be an underlying mechanisms of the associations observed, since epinephrine is a potent stimulator of ketogenesis. Interestingly, Hurr et al. demonstrated in mice that chronic hepatic sympathetic overactivity mediates hepatic steatosis and that ablation of liver sympathetic nerves was associated with improvements in liver triglyceride accumulation pathways including free fatty acid uptake and de novo lipogenesis.^52^ It could be hypothesized that an increased sympathetic tone in NAFLD may contribute to both higher circulating ketones and adverse outcomes. However, in this study we were unable to explore this hypothesis, as data on circulating catecholamines or sympathetic tone were not available.

Several methodological aspects of the current study need to be mentioned. An elevated FLI was chosen as a proxy of suspected NAFLD. The FLI is considered to have sufficient accuracy for NAFLD assessment, and its use conforms to international guidelines to apply biomarker scores in order to characterize NAFLD in larger‐sized cohorts^4^, ^5^; however, the FLI seems to perform best in European subjects.^5^ Noteworthy strengths of this study were the size of the study, the long‐term follow‐up and the extensive data collection, allowing for the adjustment for a large set of potential confounders. Furthermore, in this study ketone bodies were determined using a sensitive, precise and accurate NMR spectroscopy assay.^24^ Several limitations of this study need to be addressed as well. First, due to the observational design of this study, it is not possible to determine whether the relationships of suspected NAFLD and circulating ketone bodies with all‐cause mortality are causal or associative. Second, since BMI is part of the FLI equation, we considered it inappropriate to adjust for BMI in assessing the association of ketone bodies with suspected NAFLD in the main analyses. However, the association of ketone bodies with BMI was very modest averaging 26.0 and 26.9 kg/m^2^ in the lowest and highest ketone body tertile, respectively, and the association of an elevated FLI with ketone bodies remained essentially unaltered after adjustment for plasma insulin. Moreover, the association of total ketone bodies with an elevated FLI also remained significant in sensitivity analyses where we adjusted for age, sex and BMI or waist circumference. Third, given the epidemiological nature of our study we were unable to precisely determine the underlying mechanisms of the associations observed. Fourth, the majority of participants in the PREVEND cohort are of North European origin. Therefore, the findings cannot be extrapolated per se to people of non‐White ethnicities. Fifth, despite the finding that the association of both suspected NAFLD and total ketone bodies with all‐cause mortality remained significant after excluding the first two years of follow‐up, as well as after adjusting for many potential confounders, the possibility of reverse causation and of residual confounding cannot be fully excluded.

In conclusion, in a large general population cohort, circulating ketone bodies were higher in participants with suspected NAFLD than participants without NAFLD. In time‐to‐event analyses, both the presence of suspected NAFLD and circulating ketone bodies were independently associated with an increased risk of all‐cause mortality. Mediation analyses suggested that the association of suspected NAFLD with all‐cause mortality is to some extent mediated by circulating ketone bodies. Future studies are warranted to define in more detail the underlying mechanisms for the observed associations.

## CONFLICT OF INTEREST

The University Medical Center Groningen received research support from Labcorp in the form of a research grant and laboratory assessments to dr. R.P.F. Dullaart and Prof. dr. S.J.L. Bakker. E. Garcia and M.A. Connelly are employees of Labcorp.

## AUTHOR CONTRIBUTIONS

All authors have substantially contributed to the manuscript design and/or revision and have approved this final version of the work. The authors have agreed to take accountability for all aspects of this study. The authors’ responsibilities were as follows: Conceptualization, AP and RPFD; Data curation, EG, MAC and EGG; Formal analysis, AP, DG and RPFD; Investigation, AP and RPFD; Methodology AP and RPFD; Supervision, SJLB and RPFD; Visualization, AP and RPFD; Writing—Original draft, AP and RPFD; Writing—Review and editing, EG, EHv.d.B., JLF‐G, EGG, DG, BDW, MAC and SJLB

## Supporting information

Supplementary Material

## Data Availability

Data described in the manuscript, code book and analytic code will be made available upon request of the editor.
