# Supplementary material for: Nonalcoholic fatty liver disease, circulating ketone bodies and all‐cause mortality in a general population‐based cohort
Source: Eur J Clin Invest. 2021 Jun 13;51(12):e13627. doi: 10.1111/eci.13627 (PMC9285047; doi:10.1111/eci.13627)
Supplement: Supplementary file 1 — Supplementary Material [file ECI-51-0-s001.docx]

**Supplementary data**


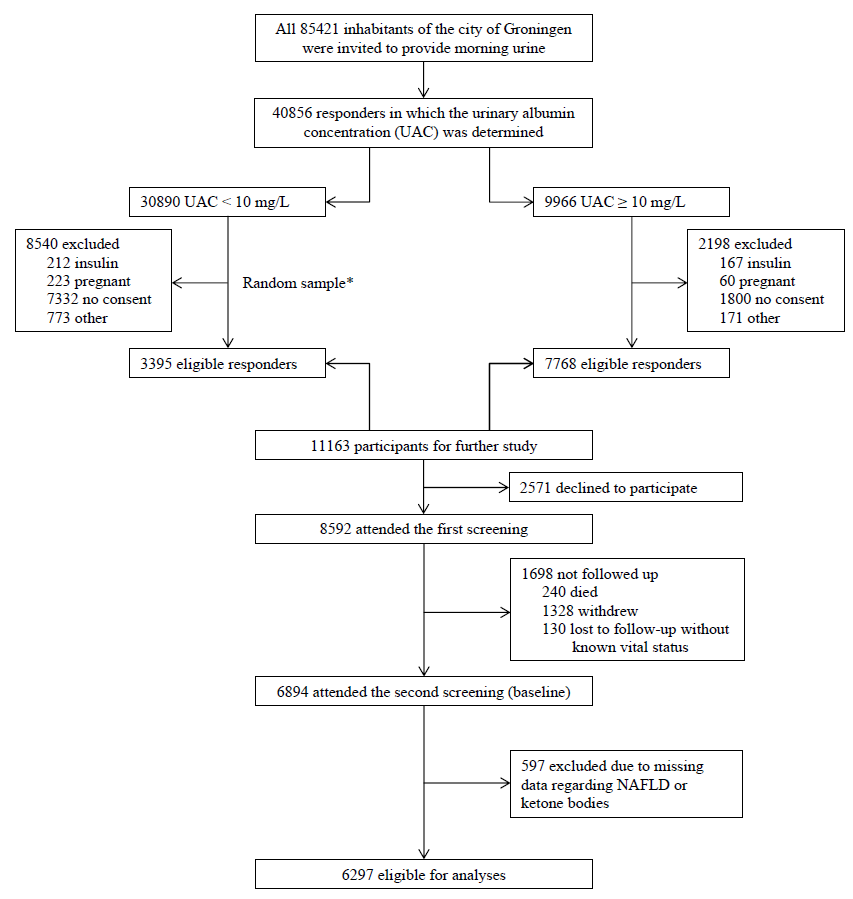


**Figure S1.** Flow of the participants through the study. *Size of the random sample was arbitrarily set at 3,395 (out of the 22,350 eligible participants) to obtain a total cohort size of approximately 10,000, taking into account a 15% non-participation rate.

| **Table S1. Plasma ketone body ratios according to suspected NAFLD (fatty liver index (FLI) ≥ 60)** | | | |
| --- | --- | --- | --- |
| **Variables** | **No NAFLD  (FLI <60**) | **NAFLD (FLI ≥60**) | **P-value** |
| Acetone to acetoacetate ratio | 0.48 [0.32 – 0.70] | 0.51 [0.34 – 0.76] | <0.001 |
| Acetone to β-hydroxybutyric acid ratio | 0.15 [0.11 – 0.21] | 0.15 [0.11 – 0.21] | 0.13 |
| Acetone to total ketone bodies ratio | 0.10 [0.07 – 0.14] | 0.11 [0.08 – 0.14] | 0.02 |
|  | | | |

| **Table S2. Baseline clinical and laboratory characteristics according to sex-stratified tertiles of total ketone bodies** | | | | |
| --- | --- | --- | --- | --- |
| **Variables** | **Tertile 1**  ♂: <7.3  ♀: < 7.2  µmol/L | **Tertile 2**  ♂: 7.3 – 7.8  ♀: 7.2 – 7.8  µmol/L | **Tertile 3**  ♂: > 7.8  ♀: > 7.8  µmol/L | **P-value** |
| Participants, n | 2099 | 2098 | 2100 | <0.001 |
| Sex, n (%) male | 1043 (50) | 1042 (50) | 1043 (50) | <0.001 |
| Age, years | 51 ± 11 | 54 ± 12 | 56 ± 12 | <0.001 |
| Type 2 diabetes, n (%) | 43 (2) | 122 (6) | 218 (10) | <0.001 |
| Hypertension, n (%) | 534 (25) | 748 (36) | 816 (39) | <0.001 |
| History of cardiovascular disease, n (%) | 106 (5) | 132 (6) | 153 (7) | 0.01 |
| History of malignancy, n (%) | 121 (6) | 122 (6) | 110 (5) | 0.65 |
| BMI, kg/m^2^ | 26.0 ± 3.9 | 27.1 ± 4.4 | 26.9 ± 4.7 | <0.001 |
| Waist circumference, cm | 90 ± 12 | 93 ± 13 | 93 ± 14 | <0.001 |
| Systolic blood pressure, mmHg | 123 ± 17 | 126 ± 18 | 128 ± 20 | <0.001 |
| Diastolic blood pressure, mmHg | 72 ± 9 | 74 ± 9 | 74 ± 9 | <0.001 |
| NT-ProBNP, ng/L | 37 [19 – 70] | 41 [21 – 81] | 48 [24 – 97] | <0.001 |
| Antihypertensive treatment, n (%) | 298 (16) | 449 (24) | 486 (26) | <0.001 |
| Smoking status, current n (%) | 581 (28) | 572 (28) | 601 (29) | 0.59 |
| Alcohol intake |  |  |  |  |
| No, almost never, n (%) | 535 (26) | 519 (25) | 521 (25) | <0.001 |
| <1 drink per day, n (%) | 1061 (51) | 1043 (50) | 917 (44) |  |
| ≥1 drink per day, n (%) | 483 (23) | 519 (25) | 639 (31) |  |
| Total cholesterol, mmol/L | 5.4 ± 1.0 | 5.5 ± 1.0 | 5.4 ± 1.1 | 0.01 |
| HDL cholesterol, mmol/L | 1.3 ± 0.3 | 1.2 ± 0.3 | 1.3 ± 0.3 | <0.001 |
| Triglycerides, mmol/L | 1.02 [0.74 – 1.43] | 1.23 [0.88 – 1.73] | 1.14 [0.83 – 1.68] | <0.001 |
| Lipid lowering drugs, n (%) | 115 (6) | 188 (10) | 195 (10) | <0.001 |
| ALT, U/L | 20 ± 13 | 21 ± 15 | 21 ± 14 | <0.001 |
| AST, U/L | 23 ± 7 | 24 ± 9 | 25 ± 11 | <0.001 |
| Gamma-GT, U/L | 21 [15 – 33] | 25 [17 – 40] | 26 [17 – 42] | <0.001 |
| Glucose, mmol/L | 4.7 [4.4 – 5.2] | 4.8 [4.5 – 5.4] | 4.8 [4.4 – 5.4] | <0.001 |
| Insulin, mU/L | 7.3 [5.3 – 10.4] | 9.1 [6.3 – 13.4] | 8.3 [5.8 – 12.8] | <0.001 |
| Glucose lowering drugs, n (%) | 16 (1) | 77 (4) | 131 (6) | <0.001 |
| eGFR, ml/min/1.73m^2^ | 95 ± 16 | 92 ± 17 | 90 ± 18 | <0.001 |
| Urinary albumin excretion, mg/24-h | 8 [6 – 13] | 9 [6 – 17] | 9 [6 – 19] | <0.001 |
| Comparisons of baseline characteristics across tertiles of total ketone bodies were tested using one-way ANOVA, Kruskal-Wallis H Test, or Chi-Square test where appropriate. | | | | |

| **Table S3.** Multivariable logistic regression analyses of ketone bodies and suspected NAFLD (fatty liver index (FLI) ≥60) in which adjustments were made for waist circumference and body mass index. | | | | | | | | |
| --- | --- | --- | --- | --- | --- | --- | --- | --- |
|  | **Total ketone bodies** | | **β-hydroxybutyric acid** | | **Acetoacetate** | | **Acetone** | |
|  | **Odds ratio [95% CI]** | **P-value** | **Odds ratio [95% CI]** | **P-value** | **Odds ratio [95% CI]** | **P-value** | **Odds ratio [95% CI]** | **P-value** |
| **Model 1** | 1.21 [1.12 – 1.31] | <0.001 | 1.07 [1.00 – 1.13] | 0.04 | 1.03 [0.97 – 1.09] | 0.3 | 1.12 [1.06 – 1.18] | <0.001 |
| **Model 2** | 1.21 [1.07 – 1.36] | 0.002 | 1.00 [0.91 – 1.09] | 0.9 | 1.01 [0.92 – 1.10] | 0.9 | 1.17 [1.08 – 1.27] | <0.001 |
| **Model 3** | 1.29 [1.15 – 1.45] | <0.001 | 1.03 [0.95 – 1.13] | 0.5 | 1.03 [0.95 – 1.12] | 0.5 | 1.21 [1.12 – 1.31] | <0.001 |
| Model 1: adjusted for age and sex. Model 2; as model 1, additionally adjusted for waist circumference. Model 3: as model 1, additionally adjusted for BMI. All ketones are log_2_-transformed for analyses. | | | | | | | | |

| **Table S4. Smoking stratified longitudinal associations of suspected NAFLD (fatty liver index (FLI) ≥60) with all-cause mortality.** | | | | |
| --- | --- | --- | --- | --- |
|  | **Current smoker** | | **No smoker** | |
|  | **HR [95% CI]** | **P-value** | **HR [95% CI]** | **P-value** |
| **Model 1** | 1.42 [0.99 – 2.05] | 0.06 | 2.47 [1.94 – 3.16] | <0.001 |
| **Model 2** | 1.07 [0.74 – 1.55] | 0.73 | 1.53 [1.19 – 1.95] | <0.001 |
| **Model 3** | 0.92 [0.60 – 1.43] | 0.73 | 1.58 [1.18 – 2.11] | 0.002 |
| **Model 4a** | 0.94 [0.61 – 1.46] | 0.80 | 1.51 [1.12 – 2.02] | 0.006 |
| **Model 4b** | 0.67 [0.41 – 1.09] | 0.10 | 1.58 [1.15 – 2.16] | 0.005 |
| **Model 5a** | 0.90 [0.58 – 1.40] | 0.65 | 1.50 [1.12 – 2.02] | 0.007 |
| **Model 5b** | 0.65 [0.40 – 1.06] | 0.08 | 1.59 [1.16 – 2.18] | 0.004 |
| Models 1: crude. Models 2: adjusted for age and sex, and alcohol usage. Models 3: as models 2, additionally adjusted for total cholesterol, HDL-cholesterol, systolic blood pressure, NT-ProBNP, eGFR and urinary albumin excretion, history of cardiovascular disease and history of malignancy. Models 4a: as models 3, additionally adjusted for type 2 diabetes. Models 4b: as models 3, additionally adjusted for glucose, insulin and use of glucose lowering drugs. Model 5a: as models 4a, additionally adjusted for total ketone bodies. Models 5b: as models 4b, additionally adjusted for total ketone bodies. | | | | |

| **Table S5.** **Kidney function stratified longitudinal associations of suspected NAFLD (fatty liver index (FLI) ≥60) with all-cause mortality.** | | | | |
| --- | --- | --- | --- | --- |
|  | **eGFR <78 ml/min/1.73m^2^** | | **eGFR ≥78 ml/min/1.73m^2^** | |
|  | **HR [95% CI]** | **P-value** | **HR [95% CI]** | **P-value** |
| **Model 1** | 1.52 [1.14 – 2.04] | 0.005 | 1.59 [1.17 – 2.14] | 0.003 |
| **Model 2** | 1.58 [1.18 – 2.13] | 0.002 | 1.07 [0.79 – 1.46] | 0.66 |
| **Model 3** | 1.75 [1.26 – 2.43] | <0.001 | 1.08 [0.76 – 1.54] | 0.66 |
| **Model 4a** | 1.72 [1.24 – 2.40] | 0.001 | 1.03 [0.73 – 1.47] | 0.86 |
| **Model 4b** | 1.60 [1.12 – 2.74] | 0.009 | 1.03 [0.73 – 1.47] | 0.86 |
| **Model 5a** | 1.70 [1.22 – 2.37] | 0.002 | 1.02 [0.71 – 1.45] | 0.92 |
| **Model 5b** | 1.58 [1.11 – 2.25] | 0.01 | 1.04 [0.70 – 1.54] | 0.86 |
| Models 1: crude. Models 2: adjusted for age and sex, current smoking and alcohol usage. Models 3: as models 2, additionally adjusted for total cholesterol, HDL-cholesterol, systolic blood pressure, NT-ProBNP, eGFR and urinary albumin excretion, history of cardiovascular disease and history of malignancy. Models 4a: as models 3, additionally adjusted for type 2 diabetes. Models 4b: as models 3, additionally adjusted for glucose, insulin and use of glucose lowering drugs. Model 5a: as models 4a, additionally adjusted for total ketone bodies. Models 5b: as models 4b, additionally adjusted for total ketone bodies. | | | | |

| **Table S6.** **NT-ProBNP stratified longitudinal associations of suspected NAFLD (fatty liver index (FLI) ≥60) with all-cause mortality.** | | | | |
| --- | --- | --- | --- | --- |
|  | **NT-ProBNP <246 ng/L** | | **NT-ProBNP**  **≥ 246 ng/L** | |
|  | **HR [95% CI]** | **P-value** | **HR [95% CI]** | **P-value** |
| **Model 1** | 1.88 [1.48 – 2.37] | <0.001 | 1.78 [1.21 – 2.62] | 0.003 |
| **Model 2** | 1.25 [0.99 – 1.59] | 0.06 | 1.62 [1.08 – 2.41] | 0.02 |
| **Model 3** | 1.18 [0.89 – 1.56] | 0.25 | 1.82 [1.13 – 2.93] | 0.01 |
| **Model 4a** | 1.13 [0.85 – 1.49] | 0.41 | 1.77 [1.09 – 2.88] | 0.02 |
| **Model 4b** | 1.08 [0.80 – 1.47] | 0.61 | 1.63 [0.97 – 2.74] | 0.06 |
| **Model 5a** | 1.10 [0.83 – 1.46] | 0.49 | 1.74 [1.07 – 2.84] | 0.03 |
| **Model 5b** | 1.08 [0.79 – 1.46] | 0.65 | 1.63 [0.97 – 2.75] | 0.06 |
| Models 1: crude. Models 2: adjusted for age and sex, current smoking and alcohol usage. Models 3: as models 2, additionally adjusted for total cholesterol, HDL-cholesterol, systolic blood pressure, NT-ProBNP, eGFR and urinary albumin excretion, history of cardiovascular disease and history of malignancy. Models 4a: as models 3, additionally adjusted for type 2 diabetes. Models 4b: as models 3, additionally adjusted for glucose, insulin and use of glucose lowering drugs. Model 5a: as models 4a, additionally adjusted for total ketone bodies. Models 5b: as models 4b, additionally adjusted for total ketone bodies. | | | | |

| **Table S7.** **History of malignancy stratified longitudinal associations of total ketone bodies with all-cause mortality.** | | | | |
| --- | --- | --- | --- | --- |
|  | **History of malignancy** | | **No history of malignancy** | |
|  | **HR [95% CI]** | **P-value** | **HR [95% CI]** | **P-value** |
| **Model 1** | 2.02 [1.42 – 2.87] | <0.001 | 1.56 [1.37 – 1.78] | <0.001 |
| **Model 2** | 1.94 [1.27 – 2.96] | 0.002 | 1.27 [1.10 – 1.47] | 0.001 |
| **Model 3** | 2.31 [1.43 – 3.76] | <0.001 | 1.24 [1.07 – 1.44] | 0.005 |
| **Model 4a** | 2.28 [1.39 – 3.72] | 0.001 | 1.21 [1.04 – 1.41] | 0.01 |
| **Model 4b** | 2.18 [1.30 – 3.64] | 0.003 | 1.19 [1.02 – 1.39] | 0.02 |
| **Model 5a** | 2.28 [1.39 – 3.72] | 0.001 | 1.20 [1.03 – 1.40] | 0.02 |
| **Model 5b** | 2.19 [1.31 – 3.67] | 0.003 | 1.19 [1.02 – 1.39] | 0.03 |
| Models 1: crude. Models 2: adjusted for age and sex, current smoking and alcohol usage. Models 3: as models 2, additionally adjusted for total cholesterol, HDL-cholesterol, systolic blood pressure, NT-ProBNP, eGFR and urinary albumin excretion, and history of cardiovascular disease. Models 4a: as models 3, additionally adjusted for type 2 diabetes. Models 4b: as models 3, additionally adjusted for glucose, insulin and use of glucose lowering drugs. Model 5a: as models 4a, additionally adjusted for either suspected NAFLD. Models 5b: as models 4b, additionally adjusted for suspected NAFLD.  Data presented per doubling of total ketone bodies. | | | | |

| **Table S8.** **Kidney function stratified longitudinal associations of total ketone bodies with all-cause mortality.** | | | | |
| --- | --- | --- | --- | --- |
|  | **eGFR <78 ml/min/1.73m^2^** | | **eGFR >78 ml/min/1.73m^2^** | |
|  | **HR [95% CI]** | **P-value** | **HR [95% CI]** | **P-value** |
| **Model 1** | 1.45 [1.20 – 1.76] | <0.001 | 1.55 [1.31 – 1.85] | <0.001 |
| **Model 2** | 1.42 [1.16 – 1.73] | <0.001 | 1.30 [1.07 – 1.57] | 0.007 |
| **Model 3** | 1.41 [1.14 – 1.74] | 0.002 | 1.27 [1.04 – 1.54] | 0.02 |
| **Model 4a** | 1.40 [1.13 – 1.73] | 0.002 | 1.23 [1.01 – 1.50] | 0.04 |
| **Model 4b** | 1.39 [1.12 – 1.74] | 0.003 | 1.21 [0.99 – 1.48] | 0.07 |
| **Model 5a** | 1.39 [1.12 – 1.73] | 0.003 | 1.23 [1.00 – 1.50] | 0.04 |
| **Model 5b** | 1.40 [1.12 – 1.74] | 0.003 | 1.22 [0.99 – 1.49] | 0.06 |
| Models 1: crude. Models 2: adjusted for age and sex, current smoking and alcohol usage. Models 3: as models 2, additionally adjusted for total cholesterol, HDL-cholesterol, systolic blood pressure, NT-ProBNP, eGFR and urinary albumin excretion, history of cardiovascular disease and history of malignancy. Models 4a: as models 3, additionally adjusted for type 2 diabetes. Models 4b: as models 3, additionally adjusted for glucose, insulin and use of glucose lowering drugs. Model 5a: as models 4a, additionally adjusted for either suspected NAFLD. Models 5b: as models 4b, additionally adjusted for suspected NAFLD.  Data presented per doubling of total ketone bodies. | | | | |

| **Table S9.** **Prospective associations of individual ketone bodies and all-cause mortality.** | | | | | | |
| --- | --- | --- | --- | --- | --- | --- |
|  | **β-hydroxybutyric acid**  **(Per doubling)** | | **Acetoacetate**  **(Per doubling)** | | **Acetone**  **(Per doubling)** | |
|  | **HR [95% CI]** | **P-value** | **HR [95% CI]** | **P-value** | **HR [95% CI]** | **P-value** |
| **Model 1** | 1.60 [0.63 – 1.80] | <0.001 | 1.31 [1.18 – 1.46] | <0.001 | 1.15 [1.04 – 1.26] | 0.006 |
| **Model 2** | 1.34 [1.18 – 1.53] | <0.001 | 1.14 [1.02 – 1.27] | 0.02 | 1.03 [0.94 – 1.14] | 0.51 |
| **Model 3** | 1.35 [1.18 – 1.55] | <0.001 | 1.17 [1.05 – 1.31] | 0.006 | 1.04 [0.93 – 1.15] | 0.51 |
| **Model 4a** | 1.32 [1.15 – 1.52] | <0.001 | 1.16 [1.04 – 1.30] | 0.01 | 1.03 [0.93 – 1.14] | 0.58 |
| **Model 4b** | 1.30 [1.13 – 1.50] | <0.001 | 1.14 [1.02 – 1.28] | 0.02 | 1.02 [0.92 – 1.13] | 0.70 |
| **Model 5a** | 1.31 [1.14 – 1.51] | <0.001 | 1.16 [1.03 – 1.30] | 0.009 | 1.02 [0.92 – 1.13] | 0.71 |
| **Model 5b** | 1.29 [1.12 – 1.49] | <0.001 | 1.15 [1.03 – 1.29] | 0.02 | 1.02 [0.92 – 1.13] | 0.75 |
| Models 1: crude. Models 2: adjusted for age and sex, current smoking and alcohol usage. Models 3: as models 2, additionally adjusted for total cholesterol, HDL-cholesterol, systolic blood pressure, NT-ProBNP, eGFR and urinary albumin excretion, history of cardiovascular disease and history of malignancy. Models 4a: as models 3, additionally adjusted for type 2 diabetes. Models 4b: as models 3, additionally adjusted for glucose, insulin and use of glucose lowering drugs. Model 5a: as models 4a, additionally adjusted for either NAFLD. Models 5b: as models 4b, additionally adjusted for suspected NAFLD.  Data presented per doubling of total ketone bodies. | | | | | | |

| **Table S10.** **Prospective associations of suspected NAFLD (fatty liver index (FLI) ≥60) and ketone bodies with cardiovascular mortality** | | | | |
| --- | --- | --- | --- | --- |
|  | **Suspected NAFLD vs. no NAFLD**  **(FLI≥60 vs. FLI<60)** | | **Total ketone bodies**  **(Per doubling)** | |
|  | **HR [95% CI]** | **P-value** | **HR [95% CI]** | **P-value** |
| **Model 1** | 2.35 [1.59 – 3.48] | <0.001 | 1.82 [1.45 – 2.27] | <0.001 |
| **Model 2** | 1.55 [1.04 – 2.30] | 0.03 | 1.47 [1.13 – 1.90] | 0.004 |
| **Model 3** | 1.42 [0.90 – 2.27] | 0.14 | 1.41 [1.06 – 1.88] | 0.02 |
| **Model 4a** | 1.36 [0.86 – 2.15] | 0.19 | 1.38 [1.03 – 1.83] | 0.03 |
| **Model 4b** | 1.15 [0.70 – 1.90] | 0.57 | 1.29 [0.96 – 1.73] | 0.09 |
| **Model 5a** | 1.32 [0.82 – 2.12] | 0.24 | 1.36 [1.02 – 1.82] | 0.04 |
| **Model 5b** | 1.14 [0.68 – 1.90] | 0.61 | 1.29 [0.96 – 1.73] | 0.09 |
| Models 1: crude. Models 2: adjusted for age and sex, current smoking and alcohol usage. Models 3: as models 2, additionally adjusted for total cholesterol, HDL-cholesterol, systolic blood pressure, NT-ProBNP, eGFR and urinary albumin excretion, history of cardiovascular disease and history of malignancy. Models 4a: as models 3, additionally adjusted for type 2 diabetes. Models 4b: as models 3, additionally adjusted for glucose, insulin and use of glucose lowering drugs. Model 5a: as models 4a, additionally adjusted for either NAFLD. Models 5b: as models 4b, additionally adjusted for suspected NAFLD. | | | | |

| **Table S11.** **Prospective associations of suspected NAFLD (fatty liver index (FLI) ≥60) and ketone bodies with non-cardiovascular mortality** | | | | |
| --- | --- | --- | --- | --- |
|  | **Suspected NAFLD vs. no NAFLD**  **(FLI≥60 vs. FLI<60)** | | **Total ketone bodies**  **(Per doubling)** | |
|  | **HR [95% CI]** | **P-value** | **HR [95% CI]** | **P-value** |
| **Model 1** | 1.90 [1.51 – 2.40] | <0.001 | 1.49 [1.29 – 1.72] | <0.001 |
| **Model 2** | 1.24 [0.98 – 1.57] | 0.07 | 1.23 [1.05 – 1.43] | 0.01 |
| **Model 3** | 1.32 [1.04 – 1.67] | 0.02 | 1.25 [1.06 – 1.46] | 0.007 |
| **Model 4** | 1.35 [1.03 – 1.77] | 0.03 | 1.25 [1.06 – 1.47] | 0.007 |
| **Model 5** | 1.38 [1.05 – 1.82] | 0.02 | 1.29 [1.10 – 1.52] | 0.002 |
| **Model 6a** | 1.34 [1.02 – 1.77] | 0.04 | 1.27 [1.08 – 1.50] | 0.004 |
| **Model 6b** | 1.33 [0.99 – 1.80] | 0.06 | 1.26 [1.07 – 1.49] | 0.006 |
| **Model 7a** | 1.32 [1.00 – 1.74] | 0.05 | 1.26 [1.07 – 1.49] | 0.006 |
| **Model 7b** | 1.32 [0.98 – 1.79] | 0.07 | 1.26 [1.06 – 1.49] | 0.007 |
| Models 1: crude. Models 2: adjusted for age and sex, current smoking and alcohol usage. Models 3: as models 2, additionally adjusted for total cholesterol, HDL-cholesterol, systolic blood pressure, NT-ProBNP, eGFR and urinary albumin excretion, history of cardiovascular disease and history of malignancy. Models 4a: as models 3, additionally adjusted for type 2 diabetes. Models 4b: as models 3, additionally adjusted for glucose, insulin and use of glucose lowering drugs. Model 5a: as models 4a, additionally adjusted for either NAFLD. Models 5b: as models 4b, additionally adjusted for suspected NAFLD. | | | | |

| **Table S12.** **Prospective associations of suspected NAFLD (fatty liver index (FLI) ≥60) and ketone bodies with cancer mortality** | | | | |
| --- | --- | --- | --- | --- |
|  | **Suspected NAFLD vs. no NAFLD**  **(FLI≥60 vs. FLI<60)** | | **Total ketone bodies**  **(Per doubling)** | |
|  | **HR [95% CI]** | **P-value** | **HR [95% CI]** | **P-value** |
| **Model 1** | 1.76 [1.32 – 2.34] | <0.001 | 1.48 [1.25 – 1.77] | <0.001 |
| **Model 2** | 1.23 [0.92 – 1.64] | 0.17 | 1.24 [1.03 – 1.51] | 0.03 |
| **Model 3** | 1.18 [0.84 – 1.65] | 0.35 | 1.31 [1.08 – 1.61] | 0.008 |
| **Model 4a** | 1.16 [0.82 – 1.63] | 0.40 | 1.31 [1.07 – 1.60] | 0.01 |
| **Model 4b** | 1.18 [0.82 – 1.72] | 0.37 | 1.30 [1.06 – 1.59] | 0.01 |
| **Model 5a** | 1.14 [0.81 – 1.60] | 0.46 | 1.30 [1.06 – 1.59] | 0.01 |
| **Model 5b** | 1.17 [0.81 – 1.71] | 0.40 | 1.29 [1.06 – 1.59] | 0.01 |
| Models 1: crude. Models 2: adjusted for age and sex, current smoking and alcohol usage. Models 3: as models 2, additionally adjusted for total cholesterol, HDL-cholesterol, systolic blood pressure, NT-ProBNP, eGFR and urinary albumin excretion, history of cardiovascular disease and history of malignancy. Models 4a: as models 3, additionally adjusted for type 2 diabetes. Models 4b: as models 3, additionally adjusted for glucose, insulin and use of glucose lowering drugs. Model 5a: as models 4a, additionally adjusted for either NAFLD. Models 5b: as models 4b, additionally adjusted for suspected NAFLD. | | | | |

| **Table S13.** **Prospective associations of suspected NAFLD (fatty liver index (FLI) ≥60) and ketone bodies with all-cause mortality after excluding participants with type 2 diabetes at baseline** | | | | |
| --- | --- | --- | --- | --- |
|  | **Suspected NAFLD vs. no NAFLD**  **(FLI≥60 vs. FLI<60)** | | **Total ketone bodies**  **(Per doubling)** | |
|  | **HR [95% CI]** | **P-value** | **HR [95% CI]** | **P-value** |
| **Model 1** | 1.80 [1.44 – 2.24] | <0.001 | 1.50 [1.31 – 1.71] | <0.001 |
| **Model 2** | 1.20 [0.96 – 1.51] | 0.11 | 1.24 [1.07 – 1.44] | 0.005 |
| **Model 3** | 1.22 [0.94 – 1.60] | 0.13 | 1.24 [1.06 – 1.45] | 0.007 |
| **Model 4a** | 1.22 [0.94 – 1.60] | 0.13 | 1.24 [1.06 – 1.45] | 0.007 |
| **Model 4b** | 1.26 [0.95 – 1.69] | 0.11 | 1.24 [1.06 – 1.45] | 0.007 |
| **Model 5a** | 1.21 [0.93 – 1.58] | 0.16 | 1.23 [1.06 – 1.44] | 0.008 |
| **Model 5b** | 1.26 [0.94 – 1.68] | 0.12 | 1.24 [1.06 – 1.45] | 0.008 |
| Models 1: crude. Models 2: adjusted for age and sex, current smoking and alcohol usage. Models 3: as models 2, additionally adjusted for total cholesterol, HDL-cholesterol, systolic blood pressure, NT-ProBNP, eGFR and urinary albumin excretion, history of cardiovascular disease and history of malignancy. Models 4a: as models 3. Models 4b: as models 3, additionally adjusted for glucose, insulin and use of glucose lowering drugs. Model 5a: as models 4a, additionally adjusted for either NAFLD. Models 5b: as models 4b, additionally adjusted for suspected NAFLD. | | | | |

| **Table S14.** **Prospective associations of suspected NAFLD (fatty liver index (FLI) ≥60) and ketone bodies with all-cause mortality after excluding participants with a history of cardiovascular disease** | | | | |
| --- | --- | --- | --- | --- |
|  | **Suspected NAFLD vs. no NAFLD**  **(FLI≥60 vs. FLI<60)** | | **Total ketone bodies**  **(Per doubling)** | |
|  | **HR [95% CI]** | **P-value** | **HR [95% CI]** | **P-value** |
| **Model 1** | 2.05 [1.63 – 2.57] | <0.001 | 1.58 [1.37 – 1.81] | <0.001 |
| **Model 2** | 1.41 [1.12 – 1.78] | 0.003 | 1.31 [1.12 – 1.52] | <0.001 |
| **Model 3** | 1.34 [1.02 – 1.76] | 0.03 | 1.32 [1.13 – 1.56] | <0.001 |
| **Model 4a** | 1.26 [0.96 – 1.66] | 0.10 | 1.29 [1.10 – 1.52] | 0.002 |
| **Model 4b** | 1.20 [0.89 – 1.63] | 0.23 | 1.27 [1.07 – 1.49] | 0.005 |
| **Model 5a** | 1.25 [0.95 – 1.64] | 0.12 | 1.29 [1.10 – 1.52] | 0.002 |
| **Model 5b** | 1.20 [0.89 – 1.63] | 0.23 | 1.27 [1.07 – 1.50] | 0.005 |
| Models 1: crude. Models 2: adjusted for age and sex, current smoking and alcohol usage. Models 3: as models 2, additionally adjusted for total cholesterol, HDL-cholesterol, systolic blood pressure, NT-ProBNP, eGFR and urinary albumin excretion, and history of malignancy. Models 4a: as models 3, additionally adjusted for type 2 diabetes. Models 4b: as models 3, additionally adjusted for glucose, insulin and use of glucose lowering drugs. Model 5a: as models 4a, additionally adjusted for either NAFLD. Models 5b: as models 4b, additionally adjusted for suspected NAFLD. | | | | |

| **Table S15.** **Prospective associations of suspected NAFLD (fatty liver index (FLI) ≥60) and ketone bodies with all-cause mortality after excluding participants with a BMI < 18.5 kg/m^2^** | | | | |
| --- | --- | --- | --- | --- |
|  | **Suspected NAFLD vs. no NAFLD**  **(FLI≥60 vs. FLI<60)** | | **Total ketone bodies**  **(Per doubling)** | |
|  | **HR [95% CI]** | **P-value** | **HR [95% CI]** | **P-value** |
| **Model 1** | 2.06 [1.69 – 2.51] | <0.001 | 1.56 [1.38 – 1.76] | <0.001 |
| **Model 2** | 1.39 [1.13 – 1.71] | 0.002 | 1.29 [1.13 – 1.48] | <0.001 |
| **Model 3** | 1.40 [1.10 – 1.77] | 0.006 | 1.31 [1.14 – 1.51] | <0.001 |
| **Model 4a** | 1.36 [1.07 – 1.72] | 0.01 | 1.28 [1.11 – 1.48] | <0.001 |
| **Model 4b** | 1.30 [1.00 – 1.69] | 0.05 | 1.26 [1.09 – 1.46] | 0.002 |
| **Model 5a** | 1.33 [1.05 – 1.69] | 0.02 | 1.27 [1.10 – 1.47] | 0.001 |
| **Model 5b** | 1.28 [0.99 – 1.67] | 0.06 | 1.26 [1.09 – 1.46] | 0.002 |
| Models 1: crude. Models 2: adjusted for age and sex, current smoking and alcohol usage. Models 3: as models 2, additionally adjusted for total cholesterol, HDL-cholesterol, systolic blood pressure, NT-ProBNP, eGFR and urinary albumin excretion, history of cardiovascular disease and history of malignancy. Models 4a: as models 3, additionally adjusted for type 2 diabetes. Models 4b: as models 3, additionally adjusted for glucose, insulin and use of glucose lowering drugs. Model 5a: as models 4a, additionally adjusted for either NAFLD. Models 5b: as models 4b, additionally adjusted for suspected NAFLD. | | | | |

| **Table S16.** **Prospective associations of suspected NAFLD (fatty liver index (FLI) ≥60) and ketone bodies with all-cause mortality after excluding participants with a BMI >30 kg/m^2^** | | | | |
| --- | --- | --- | --- | --- |
|  | **Suspected NAFLD vs. no NAFLD**  **(FLI≥60 vs. FLI<60)** | | **Total ketone bodies**  **(Per doubling)** | |
|  | **HR [95% CI]** | **P-value** | **HR [95% CI]** | **P-value** |
| **Model 1** | 2.23 [1.74 – 2.85] | <0.001 | 1.56 [1.36 – 1.79] | <0.001 |
| **Model 2** | 1.41 [1.10 – 1.82] | 0.008 | 1.28 [1.10 – 1.50] | 0.002 |
| **Model 3** | 1.44 [1.07 – 1.95] | 0.02 | 1.31 [1.12 – 1.55] | 0.001 |
| **Model 4a** | 1.42 [1.05 – 1.92] | 0.02 | 1.30 [1.10 – 1.53] | 0.002 |
| **Model 4b** | 1.39 [1.01 – 1.90] | 0.04 | 1.29 [1.10 – 1.52] | 0.003 |
| **Model 5a** | 1.39 [1.03 – 1.88] | 0.03 | 1.29 [1.09 – 1.52] | 0.003 |
| **Model 5b** | 1.37 [1.00 – 1.87] | 0.05 | 1.28 [1.09 – 1.52] | 0.003 |
| Models 1: crude. Models 2: adjusted for age and sex, current smoking and alcohol usage. Models 3: as models 2, additionally adjusted for total cholesterol, HDL-cholesterol, systolic blood pressure, NT-ProBNP, eGFR and urinary albumin excretion, history of cardiovascular disease and history of malignancy. Models 4a: as models 3, additionally adjusted for type 2 diabetes. Models 4b: as models 3, additionally adjusted for glucose, insulin and use of glucose lowering drugs. Model 5a: as models 4a, additionally adjusted for either NAFLD. Models 5b: as models 4b, additionally adjusted for suspected NAFLD. | | | | |

| **Table S17.** **Prospective associations of suspected NAFLD (fatty liver index (FLI) ≥60) and ketone bodies with all-cause mortality after excluding participants with an alcohol intake ≥ 1 consumption per day** | | | | |
| --- | --- | --- | --- | --- |
|  | **Suspected NAFLD vs. no NAFLD**  **(FLI≥60 vs. FLI<60)** | | **Total ketone bodies**  **(Per doubling)** | |
|  | **HR [95% CI]** | **P-value** | **HR [95% CI]** | **P-value** |
| **Model 1** | 1.97 [1.55 – 2.49] | <0.001 | 1.57 [1.36 – 1.81] | <0.001 |
| **Model 2** | 1.32 [1.04 – 1.67] | 0.02 | 1.29 [1.10 – 1.51] | 0.002 |
| **Model 3** | 1.25 [0.95 – 1.65] | 0.11 | 1.32 [1.12 – 1.56] | 0.001 |
| **Model 4a** | 1.23 [0.93 – 1.62] | 0.15 | 1.30 [1.10 – 1.55] | 0.002 |
| **Model 4b** | 1.21 [0.89 – 1.63] | 0.23 | 1.28 [1.07 – 1.51] | 0.005 |
| **Model 5a** | 1.21 [0.92 – 1.60] | 0.17 | 1.30 [1.10 – 1.54] | 0.002 |
| **Model 5b** | 1.21 [0.90 – 1.64] | 0.21 | 1.28 [1.08 – 1.52] | 0.005 |
| Models 1: crude. Models 2: adjusted for age and sex, and current smoking. Models 3: as models 2, additionally adjusted for total cholesterol, HDL-cholesterol, systolic blood pressure, NT-ProBNP, eGFR and urinary albumin excretion, history of cardiovascular disease and history of malignancy. Models 4a: as models 3, additionally adjusted for type 2 diabetes. Models 4b: as models 3, additionally adjusted for glucose, insulin and use of glucose lowering drugs. Model 5a: as models 4a, additionally adjusted for either NAFLD. Models 5b: as models 4b, additionally adjusted for suspected NAFLD. | | | | |

| **Table S18.** **Prospective associations of suspected NAFLD (fatty liver index (FLI) ≥60) and ketone bodies with all-cause mortality after excluding the first two years of follow-up** | | | | |
| --- | --- | --- | --- | --- |
|  | **Suspected NAFLD vs. no NAFLD**  **(FLI≥60 vs. FLI<60)** | | **Total ketone bodies**  **(Per doubling)** | |
|  | **HR [95% CI]** | **P-value** | **HR [95% CI]** | **P-value** |
| **Model 1** | 2.16 [1.75 – 2.67] | <0.001 | 1.49 [1.31 – 1.70] | <0.001 |
| **Model 2** | 1.49 [1.20 – 1.84] | 0.003 | 1.23 [1.06 – 1.43] | 0.006 |
| **Model 3** | 1.41 [1.10 – 1.82] | 0.006 | 1.26 [1.08 – 1.47] | 0.003 |
| **Model 4a** | 1.38 [1.07 – 1.76] | 0.01 | 1.23 [1.06 – 1.44] | 0.008 |
| **Model 4b** | 1.36 [1.04 – 1.79] | 0.03 | 1.22 [1.04 – 1.43] | 0.01 |
| **Model 5a** | 1.32 [1.02 – 1.70] | 0.03 | 1.22 [1.05 – 1.43] | 0.01 |
| **Model 5b** | 1.32 [1.00 – 1.75] | 0.05 | 1.22 [1.04 – 1.43] | 0.01 |
| Models 1: crude. Models 2: adjusted for age and sex, current smoking and alcohol usage. Models 3: as models 2, additionally adjusted for total cholesterol, HDL-cholesterol, systolic blood pressure, NT-ProBNP, eGFR and urinary albumin excretion, history of cardiovascular disease and history of malignancy. Models 4a: as models 3, additionally adjusted for type 2 diabetes. Models 4b: as models 3, additionally adjusted for glucose, insulin and use of glucose lowering drugs. Model 5a: as models 4a, additionally adjusted for either NAFLD. Models 5b: as models 4b, additionally adjusted for suspected NAFLD. | | | | |
